# Late Adherent Human Bone Marrow Stromal Cells Form Bone and Restore the Hematopoietic Microenvironment *In Vivo*

**DOI:** 10.1155/2013/790842

**Published:** 2013-04-24

**Authors:** Verônica Fernandes Vianna, Danielle Cabral Bonfim, Amanda dos Santos Cavalcanti, Marco Cury Fernandes, Suzana Assad Kahn, Priscila Ladeira Casado, Inayá Correa Lima, Samuel S. Murray, Elsa J. Brochmann Murray, Maria Eugenia Leite Duarte

**Affiliations:** ^1^Clinical and Basic Research Division, National Institute of Traumatology and Orthopaedics, Avenida Brasil 500, 20940-070 Rio de Janeiro, RJ, Brazil; ^2^Institute of Biomedical Sciences, Federal University of Rio de Janeiro, 21941-970 Rio de Janeiro, RJ, Brazil; ^3^Laboratory of Nuclear Instrumentation, COPPE, Federal University of Rio de Janeiro, 21941-970 Rio de Janeiro, RJ, Brazil; ^4^VA Greater Los Angeles Health Care System, Los Angeles, CA 90073, USA; ^5^University of California, Los Angeles, CA 90095, USA

## Abstract

Bone marrow stromal cells (BMSCs) are a valuable resource for skeletal regenerative medicine because of their osteogenic potential. In spite of the very general term “stem cell,” this population of cells is far from homogeneous, and different BMSCs clones have greatly different phenotypic properties and, therefore, potentially different therapeutic potential. Adherence to a culture flask surface is a primary defining characteristic of BMSCs. We hypothesized that based on the adherence time we could obtain an enriched population of cells with a greater therapeutic potential. We characterized two populations of bone marrow-derived cells, those that adhered by three days (R-cells) and those that did not adhere by three days but did by six days (L-cells). Clones derived from L-cells could be induced into adipogenic, chondrogenic, and osteogenic differentiation *in vitro*. L-cells appeared to have greater proliferative capacity, as manifested by larger colony diameter and clones with higher CD146 expression. Only clones from L-cells developed bone marrow stroma *in vivo*. We conclude that the use of late adherence of BMSCs is one parameter that can be used to enrich for cells that will constitute a superior final product for cell therapy in orthopedics.

## 1. Introduction 

In orthopedics there is an increasing demand for reproducible regeneration of bone and musculoskeletal tissues, particularly in cases of extensive tissue loss that occurs as a consequence of trauma or tumor resection. In these scenarios the use of cell therapy, associated or not with biocompatible scaffolds, has been proposed as a promising alternative to extensive surgical procedures, aiming to improve endogenous repair [1].

In humans, hematopoiesis is defined by the formation of blood cells exclusively in the bone marrow compartment in normal conditions. The effectiveness of this process depends on the presence of supporting stroma that provides a structural microenvironment for the cells and functions as a reservoir for essential growth factors [2]. In addition to its role in the homeostasis of the bone marrow, bone marrow stroma also actively participates in the maintenance and control of the functions of bone tissue by housing the osteoprogenitor cells that will differentiate in mature osteoblasts [2, 3]. 

Bone marrow stromal cells (BMSCs), also known as *mesenchymal stem cells* [4], have long been recognized as inherent osteoprogenitors [5, 6]. In 1963, Petrakova observed that the introduction of whole bone marrow fractions under the kidney capsule of mice resulted in the formation of heterotopic bone [7]. However, the identification of the putative cell population responsible for bone formation was only achieved a few years later with the pioneering studies of Friedenstein et al. [8]. Clonogenic cells with fibroblastoid morphology that demonstrated the capacity to adhere to plastic surfaces were isolated from bone marrow suspensions and proved to form bone when reintroduced *in vivo *[3, 8, 9]. Nonetheless, the biological potential of BMSCs for applications in bone repair only gained popularity after the work of Pittenger et al. [10]. Subsequently, the process of the isolation and characterization of bone marrow stromal cells became a subject of great interest in regenerative medicine.

Besides the fundamental property of adhesion to culture flasks, human BMSCs are characterized, at present, by the ability to differentiate into osteoblasts, adipocytes, and chondroblasts under standard conditions *in vitro* and to express a panel of specific surface antigens, including CD105, CD73, CD90, CD106, and CD146 [10, 12]. However, it is recognized that the isolation method based on adherence does not result in a homogenous cell population. Proliferation rate and multipotential differentiation capacity dramatically differ between initial BMSCs colonies [10, 13]. Also, many of these cell surface markers are expressed ubiquitously by other cell types such as reticular stromal cells and endothelial cells [1, 14]. Thus, while these characteristics serve to standardize a general population of BMSCs, they do not specifically define the true *stromal (or skeletal) *[15] *stem cell*, whose identity remains unknown. BMSCs populations obtained by the current protocols are believed to be, in fact, a mixture of stromal and osteoprogenitor cells in various differentiation stages [16–18], in which a subpopulation of stem cells are dispersed. From an application point of view, an optimal population of BMSCs would be one in which the majority of cells had multipotential capacity; had a high proliferative rate in order to avoid extensive *in vitro* expansion; and had the ability to form hematopoiesis-supporting stroma which is required for bone turnover [1]. 

Through the classic BMSCs isolation protocols, cells are recovered from the fraction that adheres at 3-4 days of culturing [8, 10]. However, recent reports have shown that a fraction of the remaining, nonadherent bone marrow cells, are multipotent and have self-renewal potential [19–22]. This slower adherent subpopulation has been shown to constitute 35% of the total initial population of bone marrow cells [19] and thus represents a valuable additional source of cells for therapeutic use. The possibility of obtaining a population of BMSCs, with a higher number of cells in the early stages of the isolation procedure and, mainly, enriched with cells with a major “*stemness*” state, would improve cell therapy protocols for orthopedics application. 

We hypothesized, therefore, that adherent cells from bone marrow could be fractionated in order to obtain different subpopulations of BMSCs. We compared the “classic” population, designated here as rapidly adherent BMSCs (R-cells), and late adherent BMSCs (L-cells), isolated by replating the nonadherent cells for additional three days. These two populations of cells, characterized *in vitro* and *in vivo*, were shown to have different properties, with the L-cells subpopulation presenting a high proliferation rate and the ability to reconstitute the hematopoietic microenvironment when implanted *in vivo*. 

## 2. Material and Methods

### 2.1. Cell Source

Human bone marrow stromal cells (BMSCs) were isolated from surgical waste from hip arthroplasty (acetabulum). The samples were obtained from 20 patients (9 males and 11 females) aged 43.45 ± 11.11 (range 24–59 yrs) with no significant comorbidities. All surgical procedures were performed at the National Institute of Traumatology and Orthopedics in Rio de Janeiro, Brazil. Informed consent was obtained from all individuals after approval of the study protocol by the Institutional Ethics Committee (INTO 092/2005). 

### 2.2. Cell Isolation

Reaming debris obtained from the acetabulum was transported from the operating room to the laboratory in Iscove's modified Dulbecco's medium (IMDM; Sigma-Aldrich, St. Louis, MO) supplemented with 20% fetal bovine serum (FBS; Gibco, Grand Island, NY) at 4°C and processed within 18 hours after the surgical procedure. Under sterile conditions, the whole marrow was resuspended in Ca^++^-Mg^++^-free phosphate-buffered saline (CMF-PBS), homogenized, and then allowed to stand for 30 seconds to enable sedimentation of bone spicules. The supernatant was collected and transferred to a 50 mL centrifuge tube. This procedure was repeated three times. Cell suspensions were spun 5 min at 836 ×g at 4°C, and after discarding the supernatant the pellet was resuspended in 50 mL of IMDM + 20% FBS. Cells were counted using a Neubauer chamber.

### 2.3. Rapid (R) and Late (L) Adherent BMSCs Cultures

To establish a two-stage isolation protocol, based on the time required for BMSCs to adhere to the culture flask, the cells were plated at low density to obtain colony-forming units-fibroblast (CFU-F) [8]. Cultures were initiated by plating 2.0 × 10^5^ (4.0 × 10^4^/mL) mononucleated cells obtained from the bone marrow suspensions in 25 cm^2^ flasks (nonclonal assays) or in 60 mm plates (clonal assays) + 5 mL IMDM + 20% FBS and grown for 3 days at 37°C in an air atmosphere with 5% CO_2_. At this point of the culture, the nonadherent cells from the supernatant were discarded and the adherent cells were designated as rapidly adhering cells or *R-cells*. Cell cultures were then washed with CMF-PBS and cultured in 5 mL of fresh medium for additional 10 days with medium changes 3 times a week. The cell population designated as late adherent cells or *L-cells* was obtained by plating 2.0 × 10^6^ (4.0 × 10^5^/mL) mononucleated cells in the same conditions as for R-cells until day 3. After that, nonadherent cells from the supernatant were collected and plated in 25 cm^2^ flasks (nonclonal assays) or in 60 mm plates (clonal assays) at a density of 8.0 × 10^4^ cells/mL in 5 mL of fresh IMDM + 20% FBS and grown for additional 3 days at 37°C in an air atmosphere with 5% CO_2_. At day 6, nonadherent cells were definitely discarded and the adherent cells were washed with CMF-PBS and cultured in 5 mL of fresh medium for additional 10 days with medium changes 3 times a week.

### 2.4. Human Fibroblasts

Human dermal fibroblasts (HDFs) at culture passage 16–20 were a generous gift of Professor Helio Dutra (University of Rio de Janeiro, Brazil). Fibroblasts were grown in IMDM, 10% FBS, 100 U/mL penicillin, and 100 U/mL streptomycin, for later use in the *in vivo* experiments (see following paragraphs).

### 2.5. Colony Number and Diameter

Colonies obtained from R-cells and L-cells were fixed with 10% buffered formaldehyde for 1 h at room temperature and stained with crystal violet. Only colonies containing, more than 50 cells were included in the counting and the values were expressed as the number of colonies relative to 1.0 × 10^6^ mononuclear cells plated. The diameter of round-shaped colonies was measured by two independent observers blinded with respect to the cell type (R-cells or L-cells) and ascertained as the greatest diameter of the colony. Coalescent colonies and those with blurred limits were not included in the measurements. The diameters of colonies were expressed as the mean ± SD (mm), obtained in three culture flasks for R-cells and L-cells populations. 

### 2.6. Adhesion versus Area Test

Mononuclear cells at the same concentration (2.0 × 10^6^ or 4.0 × 10^5^/mL) used for obtaining L-cells population were plated in 25 cm^2^ and in 175 cm^2^ culture flasks in a final volume of 5 mL and 30 mL of IMDM + 20% FBS, respectively. After 3 days, the supernatant of 25 cm^2^ flasks was replated in a new 25 cm^2^ culture flask and grown as described in Section 2.3. At this same point, three individual samples of 7 mL were collected from 175 cm^2^ culture flasks and centrifuged 5 min at 836 ×g at 4°C. After discarding 6 mL of the supernatant, the pellet was resuspended in 4 mL of fresh IMDM + 20% FBS, giving a final volume of 5 mL. This cell suspension was replated in 25 cm^2^ culture flasks for additional 10 days and stained for colonies identification as described in Section 2.5.

### 2.7. Cell Differentiation *In Vitro*

Nonclonal and clonal R-cells and L-cells populations were examined regarding their osteogenic, chondrogenic, and adipogenic differentiation potential. To induce osteogenesis in nonclonal cell expansion, 2.5 × 10^4^ R-cells and L-cells were plated per well in 24-well plates. After reaching confluence, the medium was changed to osteogenic medium (IMDM supplemented with 20% FBS, 10 mM *β*-glycerophosphate, 5 *μ*g/mL ascorbic acid 2-phosphate, and 10^−6 ^M dexamethasone), and the culture was continued for 21 days, with medium changes every 3-4 days. At the end of the experiment the cells were fixed in buffered formaldehyde for 1 h at room temperature, rinsed with distilled water 2X, incubated for 40 min in the dark with 2% silver nitrate solution (Von Kossa stain), and washed with distilled water 5X. For the identification of calcium deposits, the plates were exposed to UV light for 10 min. To induce adipogenic differentiation 2.5 × 10^4^ R-cells and L-cells were plated per well in 24-well plates and after confluence were incubated in adipogenic medium (IMDM supplemented with 20% FBS, 0.5 mM isobutylmethylxanthine, 10 *μ*M insulin, 200 *μ*M indomethacin, and 10^−6 ^ M dexamethasone) for 21 days, with medium changes every 3-4 days. Cells were then fixed in buffered formaldehyde for 1 h at room temperature, rinsed with absolute propylene glycol for 2 min, and incubated in 0.5% Oil Red O in propylene glycol solution for 20 min. Plates were then rinsed once with 85% propylene glycol solution and twice with distilled water to remove excess of stain. For induction of chondrogenic differentiation, micromass cultures were plated at a density of 1.0 × 10^5^ cells per 10 *μ*L spot in 96-well plates and allowed to attach for 3 hours at room temperature. After adhesion to the bottom of the well, the cell aggregates were incubated with 100 *μ*L of chondrogenic media (IMDM supplemented with 20% FBS, 10 ng/mL TGF-*β* 1, 6.25 *μ*g/mL insulin, 6.25 *μ*g/mL transferrin, and 50 *μ*g/mL ascorbic acid 2-phosphate) for 21 days, with medium changes every 3-4 days. Cell aggregates were then gently removed from the culture plates, fixed in 4% paraformaldehyde, dehydrated in a graded series of ethanol, cleared in xylene, and embedded in paraffin. Four micra sections were stained with Alcian Blue (1% Alcian Blue dissolved in 3% acetic acid, pH 2.5) for the detection of sulfated proteoglycans, characteristic of cartilaginous matrix.

For clonal assays, at day 13 for R-cells or day 16 for L-cells, cultures containing >10–30 colonies/plate, separated by a distance ≥2 round-shaped colony diameters, were washed in CMF-PBS and recovered individually. Briefly, cloning cylinders (Corning Pyrex Cloning Cylinders, 10 mm, Corning, NY) were attached to the dish with silicon vacuum grease, embracing an individual cell colony. Cells inside the cylinder were recovered with trypsin, replated in 48-well plates, and cultured until confluence (culture passage 1). Then, each clone was passaged consecutively to 24-well (culture passage 2) and 12-well plates (culture passage 3). After expansion, 1.0 × 10^4^ R-cells and L-cells were plated in 24-well plates until confluence and incubated with osteogenic or adipogenic differentiation medium. For chondrogenic differentiation, cell aggregates containing 1.0 × 10^5^ L-cells were incubated with chondrogenic medium and processed as described previously. 

### 2.8. Flow Cytometry

To ascertain the mesenchymal phenotype of nonclonal R-cells and L-cells populations, cells were released by treatment with trypsin, and 5.0 × 10^5^−1.0 × 10^6^ cells were labeled with specific fluorescent-conjugated antibodies for CD44, CD105, CD146, CD45, CD14, CD34, CD31 and analyzed on a FACSCanto cytometer (BD Biosciences, Franklin Lakes, NJ). For the clonal assay, between 13 and 16 days, cell cultures with >10–30 colonies/plate, separated by a distance ≥2 round-shaped colony diameters, were washed in CMF-PBS and recovered with trypsin as described previously in Section 2.7. Individual colonies were labeled with specific fluorescent-conjugated antibodies for CD105 and CD146 and analyzed on a FACSCanto cytometer.

### 2.9. Rapid (R) and Late (L) BMSCs Seeding on HA/TCP Vehicles

Nonclonal BMSCs from the second culture passage were used for the cellularization of hydroxyapatite/tricalciumphosphate (HA/TCP) vehicle. Cells were obtained from two donors (a female and a male aged 37 and 49 years, resp.) and each cultured separately to obtain R-cells and L-cells populations used in the implants. Briefly, 1.5 × 10^6^ R-cells and L-cells were released by treatment with trypsin, pelleted, and resuspended in 1 mL of IMDM. The cell suspensions were mixed with 30 mg of HA/TCP ceramic powder with an average particle size ranging from 65 to 100 *μ*m (morselized from Osteoset T, Wright Medical, Arlington, TN). The powder was mixed with the cell suspension and incubated at 37°C for 90–100 minutes under slow rotation (25 rpm). After a brief centrifugation, the supernatant was discarded, and the particles containing attached cells were collected and mixed with 15 *μ*L of mouse fibrinogen (3.2 mg/mL in PBS) and 15 *μ*L of mouse thrombin (25 U/mL in 2% CaCl_2_, both from Haematologic Technologies, Essex Junction, VT) forming a solid fibrin gel clot. 3.0 × 10^6^ human dermal fibroblasts (HDFs) were used in the cellularized control implant.

### 2.10. Transplantation Procedures

BALB/c nu/nu mice, aged 6–8 weeks, were purchased from the Institute of Energetic and Nuclear Research (IPEN, São Paulo, Brazil). Mice were bred and housed under specific pathogen-free conditions in the animal facility from the National Institute of Traumatology and Orthopedics. All *in vivo* procedures were performed in accordance with the regulations of the Animal Welfare Act as outlined in the National Institutes of Health Guide for the Care and Use of Laboratory Animals. Each of thirty BALB/c nu/nu mice received four subcutaneous dorsal implants containing 1.5 × 10^6^ L-cells + 30 mg HA/TCP; 1.5 × 10^6^ R-cells + 30 mg HA/TCP; 3.0 × 10^6^ HDF + 30 mg HA/TCP; and 30 mg HA/TCP. Surgeries were performed under general anesthesia achieved by intraperitoneal injection of 80–100 mg/kg ketamine hydrochloride and 10 mg/kg xylazine. Four small paravertebral incisions 10 mm in length were made on the dorsum of each mouse where single implants were placed. The incisions were closed with 5–0 mononylon simple sutures. The animals were euthanized by deep anesthesia 4 (*n* = 10), 8 (*n* = 10), and 12 (*n* = 10) weeks after transplantation. 

### 2.11. Histological Analysis of Heterotopic Bone Tissue Formation

Excised implants were fixed in 10% buffered formalin, decalcified for 5-6 weeks in 10% EDTA solution (pH 7.4), embedded in paraffin, sectioned, and stained with hematoxylin and eosin (H&E). Bone formation within the implant was estimated by two observers blinded with respect to the cell type seeded in the HA/TCP vehicle and according to the following semiquantitative scale: (0) no signs of bone formation; (1) poor bone formation restricted to a single small bone trabeculae; (2) weak bone formation; (3) moderate bone formation occupying less than one half of the section; and (4) abundant bone formation, bone spreading over more than one half of the histological section [11]. We assumed that the presence of clusters of hematopoietic cells, containing megakaryocytes and related with new bone formation, was indicative of reconstitution of the anatomy of the bone marrow microenvironment.

### 2.12. High Resolution *μ*CT

*μ*CT acquisitions were performed using a high energy spiral scan (Skyscan, model 1173, Kontich, Belgium), acquisition software version 1.6/Build 7, at 6.34 *μ*m intervals and submitted to further crosssection image reconstruction (InstaRecon CBR Server-Premium-12-8K/NRecon, version 1.6.5.8) and imaging quantification (CTAn, version 1.12.0.0). The implants were placed in a sample tube containing 10% buffered formalin inside the *μ*CT in an upright position and the number of image slices required to enclose the entire implant were obtained. All the samples were scanned using a flat panel detector (Hamamatsu, 2400 × 2400 pixels) with the same technical parameters: 90 kV at 63 *μ*A with a collected projection over 360° degrees at 0.5° angular incremental step. The exposure time for each sample was 800 ms and, in order to correct beam-hardening artifacts, an aluminum foil of 0.5 mm of thickness was used in front of the X-ray beam. Data relating bone volume to total implant volume (BV/TV) were obtained for implants displaying high bone formation (histological scores 3 and 4) and compared with noncellularized implants to confirm and quantify heterotopic *in vivo* bone formation [23]. 

### 2.13. Statistical Analysis

Quantitative data are presented as mean ± standard deviation (SD). Statistical significance of differences was evaluated by Student's *t*-test. Data were analyzed using STATA 12 (College Station, TX). P values of less than 0.05 were considered significant.

## 3. Results

### 3.1. L-Cells Have a Lower Frequency in the Bone Barrow but Have a Greater Proliferative Potential

**Figure 1:**
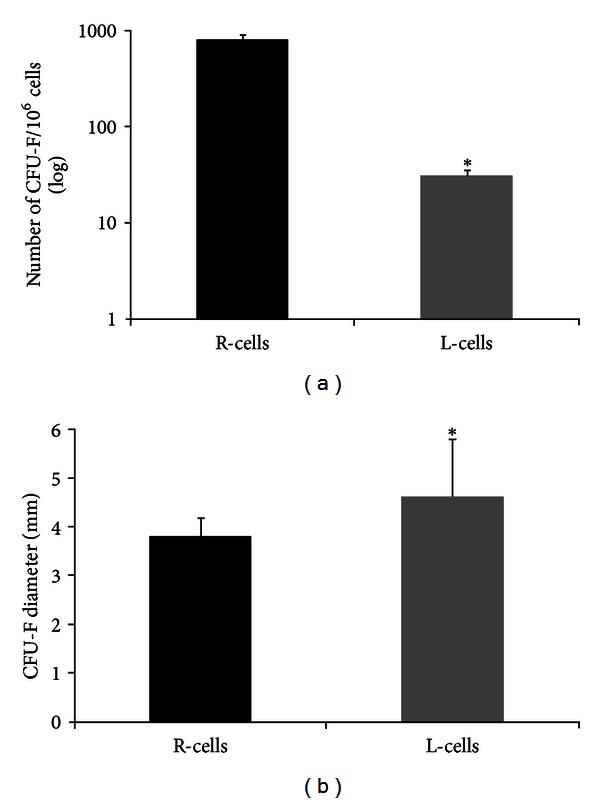
L-cells have a lower frequency in the bone marrow but a greater proliferative potential. (a) A number of colonies for R-cells (black bar) and L-cells (gray bar) populations/10^6^ mononuclear cells are represented. Results are expressed as mean ± SD, *n* = 20, *P < 0.0001. (b) Mean colony diameter for R-cells (black bar) and L-cells (gray bar) populations in mm. Results are expressed as mean ± SD, *n* = 20, *P < 0.01.

Only colonies containing more than 50 cells were counted. The final values were corrected for the initial number of mononuclear cells plated (1.0 × 10^6^) to obtain R-cells and L-cells BMSCs subpopulations. The average number of colonies formed by R-cells was significantly higher (781.25 ± 562.60, *P* < 0.0001) than the average number of colonies formed by L-cells (29.75 ± 24.41) (Figure 1(a)). The diameter of round colonies formed by R-cells and L-cells subpopulations was measured without prior knowledge of the colony cell type. Colonies with coalescent or undefined borders were excluded from the measurements. The mean diameter of colonies formed by L-cells was significantly greater (4.6 ± 1.2 mm, *P* < 0.05) than the mean diameter of colonies formed by R-cells (3.8 ± 0.4 mm) (Figure 1(b)). The ability of R-cells to form a greater number of colonies indicates that its frequency in the bone marrow is higher than L-cells. On the other hand, considering that a colony results from clonal proliferation, larger diameter of individual colonies indicates a greater cellular proliferative potential due to the initiation of the colony by a lesser committed progenitor. 

### 3.2. The Area of Cultivation Is Not a Limiting Factor for the Adhesion of BMSCs

**Figure 2:**
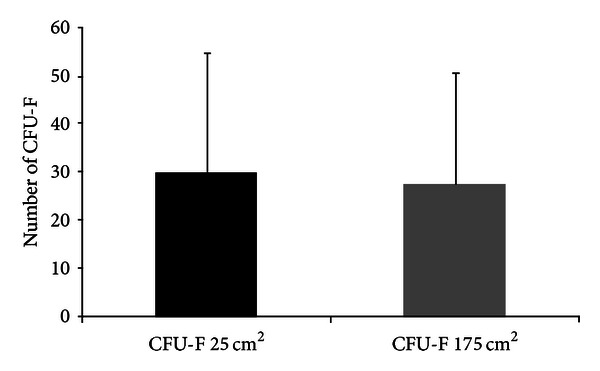
The area of culture flask is not a limiting factor for cell adhesion. Mean number of colonies for L-cells cultured in 25 cm^2^ (black bar) and 175 cm^2^ (gray bar) culture flasks for 3 days. Results are expressed as mean ± SD, *n* = 7, *P* = 0.84.

To rule out the possibility that the contact area between the cells and the plastic from the culture flask was a limiting factor for cell adhesion, we plated in 175 cm^2^ culture flasks the same initial cell concentration used to obtain L-cells in 25 cm^2^ culture flasks. After 3 days in culture, cells were replated in standard conditions (25 cm^2^) for additional 10 days. There was no difference in the number of colonies, independent of the initial area of cultivation (Figure 2). Our results demonstrate that there is a fraction of BMSCs which adheres more slowly to the culture flask denoting that this is an intrinsic biological property of this cell subpopulation and does not constitute a technical artifact.

### 3.3. L-Cells Are Multipotent Cells with Phenotypic Characteristics of BMSCs

**Figure 3:**
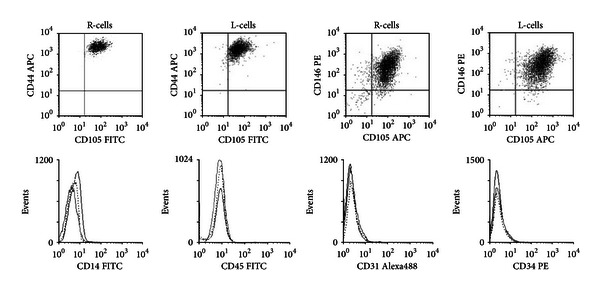
L-cells and R-cells display phenotypic characteristics of BMSCs. Multiclonal flow cytometry analysis demonstrates that both L-cells and R-cells are positive for CD44, CD105, and CD146 and negative for CD14, CD45, CD31, and CD34.

We analyzed surface markers characteristic of BMSCs [12] in R-cells and L-cells by flow cytometry. As expected, both cell populations were positive for CD44, CD105, and CD146, surface proteins known to be expressed by BMSCs, and negative for CD14, CD45, CD31, and CD34, markers of hematopoietic lineage cells (Figure 3). These data confirm the L-cells as BMSCs. 

**Figure 4:**
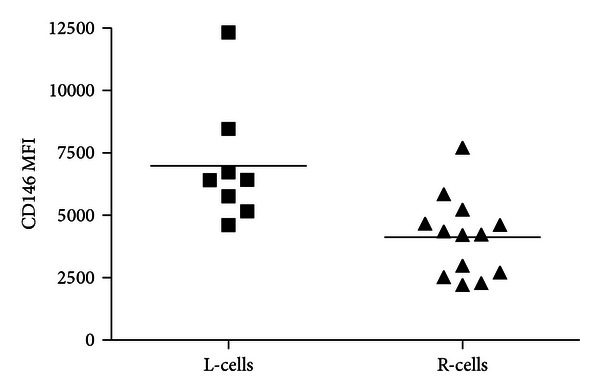
L-cells, compared to R-cells, have a higher expression of CD146. Mean fluorescence intensity (MFI) of CD146 expression in CD105^+^ L-cells and R-cells derived from individual colonies. Results are expressed as median, *n* = 3, *P* < 0.02.

The expression of CD146 is associated with cell populations displaying greater multipotentiality [14]. For this reason we compared the intensity of the expression of CD146 on individual R-cells and L-cells colonies positive for CD105. The mean fluorescence intensity of CD146 was significantly higher (*P* < 0.05) in the colonies formed by L-cells (Figure 4), suggesting that L-cells, compared to R-cells, constitute a subpopulation of BMSCs with greater multipotentiality. 

### 3.4. L-Cells Have a Trilineage Differentiation Potential *In Vitro*

**Figure 5:**
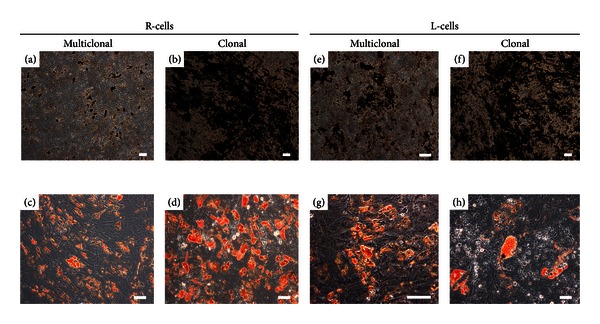
R-cells and L-cells differentiate along the osteogenic and adipogenic lineage. (a, b, e, f) Osteogenic differentiation in clonal and nonclonal R-cells and L-cells cultures, respectively. (c, d, g, h) Adipogenic differentiation in clonal and nonclonal R-cells and L-cells cultures. Scale bars = 100 *μ*m.

To demonstrate the multipotentiality of both BMSCs subpopulations, R-cells and L-cells were submitted to differentiation assays for the three major lineages of mesodermal origin (osteogenic, adipogenic and chondrogenic). Osteogenic and adipogenic differentiation potential, investigated by the method of Von Kossa and Oil Red O, respectively, was positive for R-cells and L-cells in nonclonal and clonal cell expansions (Figure 5). 

**Figure 6:**
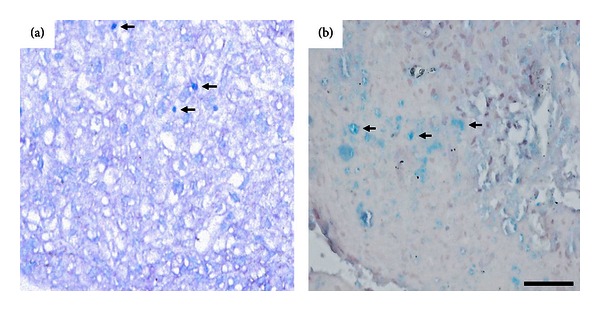
Chondrogenic differentiation of nonclonal (a) and clonal (b) L-cells. Deposits of sulfated proteoglycan matrix (arrows) are seen in both types of L-cells expansion. Scale bar = 100 *μ*m.

The chondrogenic differentiation potential was assessed in L-cells by Alcian Blue staining in a three-dimensional culture model. Deposits of sulfated proteoglycan matrix, characteristic of chondrogenic matrix, were seen in both nonclonal and clonal L-cells expansion, (Figure 6). These results confirm the trilineage differentiation potentiality of L-cells.

### 3.5. L-Cells, but Not R-Cells, Reconstituted the Hematopoietic Microenvironment *In Vivo*

**Figure 7:**
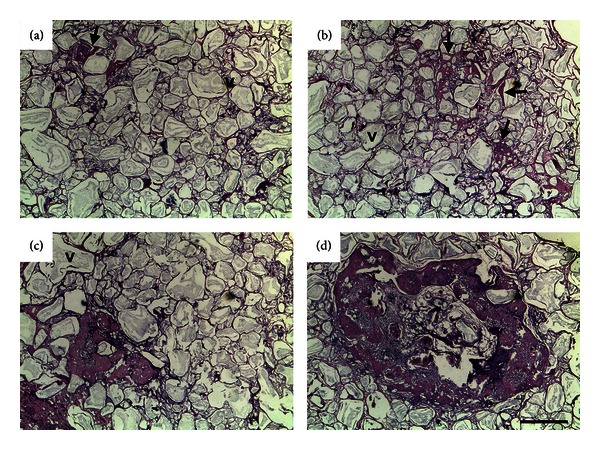
Histological grading of heterotopic bone formation in HA/TCP implants containing R-cells, L-cells, or vehicle (v) only. The following semiquantitative scale was used: (0) no signs of bone formation (not shown); (1) (panel a) poor bone formation restricted to a single small bone trabeculae (arrow); (2) (panel b) weak bone formation (arrows); (3) (panel c) moderate bone formation occupying less than one half of the section; and (4) (panel d) abundant bone formation, bone spreading over more than one half of the histological section [11]. Four micra paraffin-embedded sections stained by H&E. Scale bar = 500 *μ*m.

*In vivo* experiments were performed to evaluate the ability of R-cells and L-cells to form bone and/or other specialized tissues. Implants (*n* = 118) were assigned scores in terms of bone formation as detailed above and illustrated in Figure 7.

**Figure 8:**
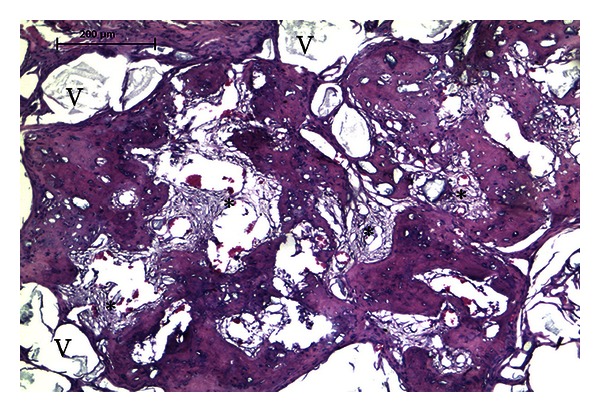
Abundant bone formation in implant loaded with R-cells harvested at 12 weeks. The spaces between the bone trabeculae are filled with fibrovascular tissue (∗), with dilated capillaries containing red blood cells. Despite the expressive bone formation with bone spreading over the histological section, there is no evidence of hematopoiesis. Residual vehicle (v) particles are seen as empty clear spaces. Four micra paraffin-embedded section stained by hematoxylin and eosin.

The pattern of the response of the two types of cells as manifested in the bone formation score was complex (Table 1). At 4 weeks more R-cells formed bone. At 8 weeks the number of implants forming any bone was similar (7/10 for R-cells versus 8/9 for L-cells) whereas at 12 weeks, more R-cells implants formed any bone (9/10 for R-cells versus 6/9 for L-cells) (Figure 8). 

**Figure 9:**
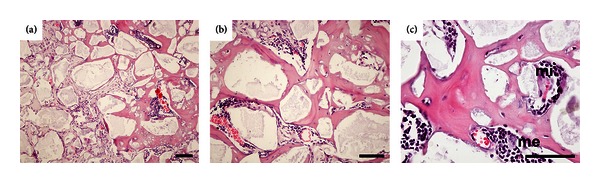
Implants loaded with L-cells. Note that the hematopoietic microenvironment is reconstituted *in vivo*. (a) Interconnecting bone formation is seen on the surface of HA/TCP particles. A small cluster of hematopoietic tissue is in close association with a blood vessel. (b) Well-organized bone deposition reproduces the classical microanatomy of cancellous bone. The hematopoietic tissue clearly shows a spatial relationship with the blood vessels and with bone. (c) Detail from the hematopoietic tissue containing cells of the erythroid (lower left), myelocytic (mi) and megakaryocytic (me) series in close association with the vascular network. Structural maturity of the bone is verified by the orderly disposition of osteocytes within the bone. Four micra paraffin-embedded sections stained by H&E. Scale bars = 100 *μ*m.

Only implants loaded with L-cells formed bone marrow stroma, the *sine qua non* of true mature bone formation and reestablishment of the bone marrow microenvironment (Figure 9). Cells from erythrocytic, myelocytic, and megakaryocytic series were observed spatially associated with bone and newly formed blood vessels (Figure 9(c)). The development of clusters of hematopoietic tissue occurred in all time points without a time-dependent relationship (1/2 in the fourth week, 3/8 after 8 weeks, and 2/6 after 12 weeks). However, only implants displaying intense bone formation (implants classified with histological scores III and IV) developed bone marrow stroma. Control implants loaded with human fibroblasts and vehicle only, harvested at 4, 8, and 12 after posttransplantation, did not show any signs of either osteogenesis or hematopoiesis. No inflammatory response of any type was observed in any of the implants or the peri-implant regions. Immunoreactivity to human laminin was observed in sections of implants featuring hematopoiesis (not shown).

### 3.6. High Resolution *μ*CT Analysis of HA/TCP Implants, Loaded with Human BMSCs, Reproduces the Findings of Quantitative Histological Analysis

**Figure 10:**
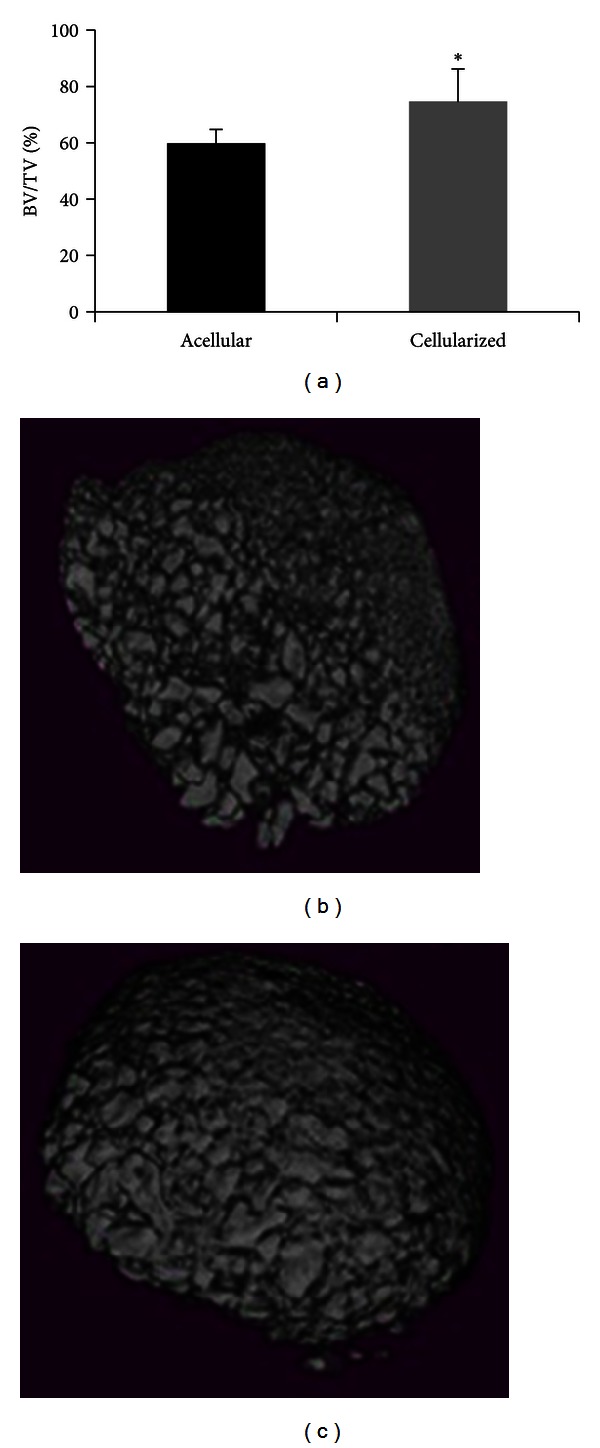
Evaluation of heterotopic bone formation by 3D *μ*CT analysis at 8 weeks. (a) Parameter relating bone volume to total implant volume (BV/TV) used to compare noncellularized and cellularized implants displaying high (≥3) histological bone formation. (b) Cross-sectional image reconstruction of noncellularized implant (BV/TV = 54.3%). (c) Cellularized implant (BV/TV = 85.7%) with exuberant histological bone formation (histological score 3). *P < 0.05.

Micro CT-analysis was performed to investigate the proportion of HA/TCP implants that was occupied by bone. After capture and reconstruction of three-dimensional radiographic images, the BV/TV index, defined by the ratio between the volume of bone tissue (mineralized) and total sample volume, was calculated in the samples with high bone formation (scores 3 and 4) based on the histological analysis. In both control groups (acellular and implants seeded with HDF), 59.6 ± 5.7% of the total volume of the sample was represented by the mineralized phase. In implants seeded with BMSCs, regardless of the cell type (R-cells or L-cells) and with abundant bone formation (histological grades III and IV), the volume of the sample represented by the mineralized phase was 74.55 ± 11.06% (*P* < 0.005) (Figure 10). The *μ*CT analysis correlated with the assignment of histological scores to the degree of bone formation in implants of HA/TCP, loaded with human BMSCs. Also, the method proved to be an effective form for identifying and quantifying the mineral phase within HA/TCP implants enriched with BMSCs and placed in the dorsum of mice for 4–12 weeks.

## 4. Discussion

Since the pioneering study of Friedenstein et al. [8], which defined the fibroblast colony-forming units (CFU-F), investigators have looked to adherent fraction of bone marrow-derived cells as a potential source of multipotent progenitors that can be induced to form bone tissue [3, 10, 11]. This cell population, however, displays a high degree of phenotypic heterogeneity, which might be a primary barrier to their use in regenerative medicine. Optimally useful cells for a clinical grade product would be a homogeneous population with a high proliferative capacity to form the mass of tissue required, that is able to be directed to complete osteogenic differentiation, and that has the capacity to form an appropriate bone marrow stroma which is required for subsequent *in vivo* turnover [1]. In an effort to develop a method to produce large numbers of cells with the characteristics stated above, we have undertaken studies to stratify human BMSCs obtained through a two-stage isolation protocol based on the time required for the cells to adhere to the culture flask surface. The studies described herein characterize the biological properties of two populations of cells; R-cells that adhere to the culture flask surface within up to three days after plating and L-cells that adhere to the surface of culture materials not after three days but by six days. The cells that adhered more slowly (L-cells) not only form bone when implanted *in vivo* but also are able to reorganize the hematopoietic microenvironment, suggesting that this subset of BMSCs is enriched with cell clones in a less committed state of differentiation. 

The first evidence that the nonadherent fraction of bone marrow suspensions were a putative source of osteoprogenitors with the potential to differentiate into osteoblasts *in vitro* was described by Long et al. [20]. The authors observed that the nonadherent cell fraction of bone marrow, cultured for 7 days under serum-free conditions, contained a population of immature cells with a limited capacity to proliferate, which contained high levels of cytoplasmic bone-related proteins. This cell population failed to respond to regulatory stimuli of hematopoietic cells, such as interleukins-1, -3, -6, and GM-CSF, but they were shown to be highly proliferative in the presence of bone-related regulators, such as TGF-*β*. Furthermore, in response to serum addition, these cells becam adherent and deposited bone proteins (osteonectin, osteocalcin, and collagens type I and type III) and calcium into the extracellular matrix, a phenotype characteristic of osteoblast-like cells [20].

Indeed, the capacity of putative osteoprogenitor cells of surviving and proliferating in suspension, under specific *in vitro* conditions, has been reinforced by Baksh et al. [24]. In order to address the regulatory roles that hematopoietic cells exert in the function and behavior of mesenchymal progenitors, both hematopoietic and nonhematopoietic populations were cocultured in a suspension system, thus limiting the effects of adhesion, once adherence to plastic, similar to that already established for hematopoietic progenitors, has been demonstrated to theoretically activate the events that induce the differentiation of mesenchymal progenitors [25]. In their results, they observed that under this coculture suspension system, the output number of mesenchymal progenitor cells, characterized by the ability to differentiate *in vitro* to the adipogenic and osteogenic pathways, was significantly greater in comparison with the number obtained through adherent cultures [24]. 

In the present study, what initially distinguished both subpopulations was the time necessary for adhesion to the culture flask. Though the property to adhere to the plastic of the culture flask is a well-defined characteristic of BMSCs, it has not yet been fully established how this property can influence the biological behavior of this cell population. The success of cell therapy, particularly for the repair of large bone defects, depends on obtaining sufficient numbers of osteogenic cells, which requires an extended time for cell culture and expansion. Many studies have focused on how to increase the effectiveness of the cultivation of BMSCs and how to extract the largest number of BMSCs from bone marrow samples. Wan et al. [21] have proposed a simple and efficient protocol to increase the number of BMSCs in a given cell culture by replating nonadherent cells from the supernatant. They demonstrated that both BMSCs populations were similar in various biological parameters. Both the originally adherent and nonadherent cells had similar proliferative and differentiation potentials *in vitro* and *in vivo*. The use of nonadherent cells in the cell expansion increased by 36.6% the number of BMSCs, which qualifies this cell fraction as a complementary source of BMSCs [21]. These results are in accordance with our finding that in the bone marrow exists a subpopulation of BMSCs with proven osteogenic potential that takes slightly longer to adhere to the plastic surface of a culture flask. 

To determine if multipotent cells are present in a certain population of BMSCs, one must perform rigorous analysis of their differentiation potential. With regard to bone regeneration, the ability to form bone, hematopoiesis-supporting stroma, and marrow adipocytes defines the multipotent nature of a CFU-F. The ability of BMSCs to establish osteogenesis and recreate marrow stroma is better established *in vivo,* after transplantation of individual colonies. However, when a series of clonal cell lineages derived from a single CFU-F are transplanted *in vivo,* only 10–20% recreate the bone/marrow organ [11]. Therefore, not all BMSCs, even not all colonies, are multipotent. By establishing a culture of BMSCs, we are not establishing a culture of multipotent cells, but rather a culture where only a fraction of the cells are multipotent [1].

Nevertheless, as shown here, the greater expression of CD146 by L-cells and the development of bone marrow stroma only in implants seeded with L-cells allow us to assume the existence of two subpopulations of BMSCs with distinct but possibly complementary, biological characteristics. With respect to proliferation, R-cells formed more colonies initially indicating a higher frequency in the bone marrow. On the other hand, L-cells colonies had a greater mean diameter indicating that on a per cell basis, L-cells had a greater proliferative capacity. Moreover, the larger size of colonies indicates, similar to what is observed in the culture of hematopoietic cells, a greater proliferative capacity and a lesser commitment to differentiation of the cell that initiated the clonal formation of the colony [26]. Both R-cells and L-cells were capable of terminal osteoblastic differentiation as manifested by formation of calcified colonies, which requires progression through all of the preceding stages of osteoblastic differentiation. The attribute of L-cells to differentiate into three mesodermal lineages (osteogenic, adipogenic, and chondrogenic) in *in vitro* clonal assays proves the trilineage potential of this cell fraction. 

The higher expression by L-cells of the surface molecule CD146 is an additional feature of this subset of BMSCs, which may constitute an advantage for its use in cell therapy protocols. The expression of CD146, a classic marker of BMSCs, is associated with the cellular ability to form bone marrow stroma and, hence, to reconstitute the hematopoietic microenvironment. The reestablishment of the microanatomy of the bone marrow in heterotopic sites derives from a sequence of events in which bone formation precedes the formation of bone marrow stroma and hematopoiesis. Cells with high levels of expression of CD146 have a high renewing potential and are able to form bone and bone marrow stroma. BMSCs with low expression of CD146 possess only the capacity to form bone [14].

The results from studies aiming to optimize the isolation of a greater number of BMSCs with osteogenic potential are in agreement with our hypothesis [19]. About 35% of the total initial number of BMSCs obtained by a fractionated isolation protocol based on the time of cell adhesion to the culture flask surface consisted of cells that required longer to adhere. Likewise both subsets of BMSCs contained CFU-Fs, able to form colonies, with the number of colonies being higher in the subset of rapid adherent cells. 

Only implants loaded with L-cells formed clusters of hematopoietic tissue together with angiogenesis and also with significant amounts of new bone formation (histological grades III and IV). This finding was observed in implants harvested at all time points (4, 8, and 12 weeks). The presence of bone and hematopoietic tissue in implants loaded with BMSCs after 8 weeks is a good indicator of the tissue organization that will be formed later [27].

The islands of hematopoietic tissue detected in the implants loaded with L-cells were spatially related to mature bone and the development of a functional vascular supply within the implant. The promotion of *in situ* angiogenesis possibly enhances bone morphogenesis through cellular and molecular interactions between endothelial populations and bone cell populations [28]. As the implant develops a vascular network, bone is formed in an orderly manner, and only when structural maturity is reached does marrow stroma develop and become colonized by hematopoietic cells. The relationship between the number of osteoblasts, bone formation, and minimal development of hematopoietic microenvironment has been described previously [22, 29].

Highresolution *μ*CT is an effective method for the analysis of bone formation *in vivo* in complex structures with reduced size such as the HA/TCP implants used here. In this study the amounts of bone demonstrated by tomography correlated well with the results of direct histological examination. 

 In conclusion, our results suggest that L-cells, obtained from the supernatant of primary bone marrow plating that is routinely discarded, constitute a natural reservoir of less committed osteoprogenitors. The use of this subset of BMSCs, for the enrichment of cell products with clinical purpose in orthopedics, may offer advantages over conventional protocols using only the rapid adherent BMSCs fraction. In the context of cell therapy strategies for bone tissue engineering, L-cells could be an alternative to provide a more suitable final cell product while reducing the manipulation in the laboratory and the time required for cell expansion. Future studies will be directed towards using the parameters described here to develop a protocol of sequential stratification that will result in a more homogeneous population of cells showing the desired characteristics. 

## Figures and Tables

**Table 1:** Histological scores for bone formation in implants cellularized with R-cells and L-cells recovered 4, 8 and 12 weeks after surgery.

Type of Implant	(*n*)	Post-implantation time (weeks)			Bone Formation Score			
			0	I	II	III	IV	Marrow Stroma (*n*)
R-cell	10	4	6	—	3	1	—	—
	10	8	3	2	3	2	—	—
	10	12	1	1	3	5	—	—

L-cell	10	4	8	—	1	1	—	1
	9	8	1	1	2	4	1	3
	9	12	3	—	2	4	—	2
